# Effect of Physical and Chemical Treatments on Viability, Sub-Lethal Injury, and Release of Cellular Components from *Bacillus clausii* and *Bacillus coagulans* Spores and Cells

**DOI:** 10.3390/foods9121814

**Published:** 2020-12-07

**Authors:** Antonio Bevilacqua, Leonardo Petruzzi, Milena Sinigaglia, Barbara Speranza, Daniela Campaniello, Emanuela Ciuffreda, Maria Rosaria Corbo

**Affiliations:** Department of Agriculture, Food, Natural Resources and Engineering (DAFNE), University of Foggia, 71122 Foggia, Italy; leonardo.petruzzi@unifg.it (L.P.); milena.sinigaglia@unifg.it (M.S.); barbara.speranza@unifg.it (B.S.); daniela.campaniello@unifg.it (D.C.); emanuela.ciuffreda@virgilio.it (E.C.); mariarosaria.corbo@unifg.it (M.R.C.)

**Keywords:** age of spores, release, proteins, DPA, injury

## Abstract

Bacterial spores are of concern to the food industry due to their ability to survive processing and their potential to subsequently germinate and grow in food. In this paper, two strains belonging to the genus *Bacillus* (*B. clausii* DSM 8716 and *B. coagulans* DSM 1) were studied under in vitro conditions after the application of essential oils, and physical treatments; cells and spores’ susceptibility, the extent of sub-lethal injury and the release of cellular components as a function of treatment and targets (cells, spores, old or activated spores) were studied. The highest antimicrobial effect was found for cells treated through citrus extract, while both essential oils and physical treatments could cause a sub-lethal injury on the surviving cells and spores; in addition, the spores of *B. coagulans* released dipicolinic acid (DPA) and proteins. Sub-lethal injury should be considered when designing a food processing treatment, because injured microorganisms could either repair the damage or be inactivated with a different effect on microbial stability of foods.

## 1. Introduction

Food contamination generally occurs on “foods that are spoiled or tainted because they either contain microorganisms, such as bacteria or parasites, or toxic substances that make them unfit for consumption” [1]. Physical and chemical methods are commonly used to control spoiling and pathogenic bacteria; after these treatments, microorganisms may be killed, survive, or be sub-lethally injured [2]. Injury is a great threat in food industry because injured microorganisms could repair themselves and return to a normal physiological state [3].

Many spoilers are spore-forming bacteria, belonging to a wide variety of genera (*Clostridium*, *Bacillus*, *Alicyclobacillus*, *Geobacillus* etc.) [4]. Bacterial spores are of concern to the food industry because they could survive treatments, then germinate and grow in food, thereby decreasing safety and shelf-life [5]. Spores, like cells, could be sub-lethally injured and this phenomenon must be considered because of the complexity of the spore entity and its intrinsic high resistance to stress. The extent of sub-lethal damage and mechanisms of injury and repair are quite variable and rely upon the conditions of stress and resuscitation [6].

Currently, thermal processing remains the principal and traditional method of microbial inactivation and reduction; there are several kinds of thermal treatments in foods, like High Temperature-Long Time (HTLT, temperature ≥ 80 °C and holding times ≥ 30 s), High Temperature-Short Time (HTST, temperature ≥ 80 °C and holding times ≤ 30 s), Mild Temperature-Long Time (MTLT, temperature < 80 °C and holding time > 30 s), and Mild Temperature-Short Time (MTST, temperature ≤ 80 °C and holding time ≤ 30 s) [7,8,9]. Thermal treatments could induce several negative effects on the physico-chemical parameters of food; moreover, some mild treatments were shown to induce a large portion of sublethal injury presenting a potential hazard to food safety [10]. 

The increasing demand for foods with a fresh-like quality (texture, color, and aroma) and the desire to produce foods with functional ingredients has increased interest in the development of low-heat operations to either inactivate spores or to prevent their germination and growth [5]. Some non-thermal pasteurization processes have been proposed during the last decades, including ultrasound (US) and high-pressure homogenization (HPH) [11,12]. Among natural products, essential oils (EOs) of higher plants and their components are gaining interest as food additives and are widely accepted by consumers because of their relatively high volatility, ephemeral, and biodegradable nature [13]. The available literature reported that EOs possess, among others, significant antiseptic, antibacterial, antiviral, antioxidant, anti-parasitic, antifungal, and insecticidal activities [14], and their bioactivity towards spores was extensively reported in the past.

*Bacillus coagulans* is a non-pathogenic, facultative anaerobic, thermotolerant, and acidophilic bacterium, with a significant spoiling impact. In acidified canned vegetable products (pH ca. 4–4.5), this microorganism is frequently found since spores can grow and germinate at pH values as low as 4. Moreover, this bacterium can increase the pH of food products to values that may allow for germination of *Clostridium botulinum* spores [15].

*Bacillus clausii* is a probiotic present on the market for over 55 years. Its resistance to both physical and chemical conditions (such as heat, antibiotics, and gastric pH) is attributed to its spores. As *B. clausii* is highly resistant to most antibiotics, its efficacy is not altered by concomitant antibiotherapy. Moreover, *B. clausii* is unique by its fast growth in aerobic and anaerobic environments [16].

The main goal of this paper was to evaluate the effect of two EOs (lemon extract and citrus extract), and three physical stresses (HPH, US, and heating) on two collection strains of *Bacillus* spp., i.e., *B. clausii* DSM 8716 and *B. coagulans* DSM 1, as models for the genus. The effect of chemical and physical treatments was studied as a function of the targets (spores, cells, activated spores) and spores’ age (fresh and old spores). Another additional goal was to investigate the occurrence of sub-lethal injury after each stress application, and the relationship between some combinations that determined a significant sub-lethal injury and the kind of damage occurring on spores.

## 2. Materials and Methods

### 2.1. Strains and Culture Conditions

*Bacillus clausii* DSM 8716 and *Bacillus coagulans* DSM 1 from Deutsche Sammlung von Mikroorganismem und Zellkulturen’s collection (DSM, Braunschweig, Germany) were used throughout this research.

*B. clausii* was stored at 4 °C on Alkaline Nutrient Agar (Nutrient Agar-Oxoid, Milan, Italy), supplemented with 10% of a sterile 1 M Na-sesquicarbonate solution (4.2 g of NaHCO_3_—Sigma-Aldrich—and 5.3 g of Na_2_CO_3_ anhydrous—Sigma-Aldrich—in 100 mL of distilled water) and 0.5% of NaCl. The microorganism was grown in Alkaline Nutrient broth with 0.5% of NaCl, and incubated for 2 days at 30 °C. *B. coagulans* was stored at 4 °C in Nutrient Agar and grown in Nutrient broth incubated for 2 days at 40 °C.

### 2.2. Spore Production

*B. coagulans* was grown in Trypticase Soy broth (Oxoid) supplemented with 0.6% Yeast Extract (Oxoid), 500 mg/l of Manganese Sulfate Monohydrate and 3 mg/l of Dextrose (Sigma-Aldrich, Milan, Italy) at 37 °C for 2 days; then, it was plated on Nutrient Agar at 50 °C for 7 days. When sporulation reached 90%, spores were harvested by adding 5 mL of sterile distilled water, detaching with sterile glass beads, collecting with a pipette and re-suspending in sterile distilled water. The suspension was centrifuged five times at 4000× *g* at 4 °C for 10 min. Heat treatment at 80 °C for 10 min was applied to destroy any remaining vegetative cells; spore suspension was stored at 4 °C. Spore number was assessed through the spread plate count on Nutrient Agar, incubated at 40 °C for 2 days.

Spores of *B. clausii* were produced on Alkaline Nutrient Agar with 0.5% of NaCl, supplemented with 10.0 mg/L of Manganese Sulfate Monohydrate (Sigma-Aldrich), incubated at 30 °C for 11 days until approximately 80–90% of cells sporulated. Spores were removed and heat-treated at 80 °C for 10 min, to eliminate vegetative cells and stored at 4 °C. Spore number was assessed through the spread plate count on Alkaline Nutrient Agar with 0.5% of NaCl, incubated at 30 °C for 2 days.

### 2.3. Chemical and Physical Treatments

#### 2.3.1. Target of the Experiments

The targets were: (i) Fresh spores: Produced and used within 2 weeks; (ii) old spores: Produced and stored at 4 °C for 4 months before the experiments; (iii) activated spores: Spores heated at 70 °C for 10 min before the analysis; (iv) cells.

#### 2.3.2. Chemical Treatments

Lemon extract (Spencer Food Industrial, Amsterdam, The Netherlands) and citrus extract (Biocitro^®^, Quinabra, Probena, Spagna) were used as the active compounds in this study. Stock solutions (25,000–50,000 ppm) (1 ppm = 1 mg/L) were freshly prepared before each use in ethanol-water (1:1, *v*/*v*) for lemon extract, and in distilled water for citrus extract, and sterilized by filtering through membranes (0.2 μm, Millipore, Milan, Italy).

#### 2.3.3. Physical Treatments

High homogenization pressure was performed for 1, 2, or 3 times at 150 MPa through a high-pressure homogenizer PANDA 2K (Niro Soavi s.p.a., Parma, Italy); the duration of each step of homogenization was 1–2 s. The circuits were cleaned with sterile distilled water (70 °C) and cooled with water (20 °C) to obtain an exit temperature of the samples of 45–50 °C. After the homogenization, samples were collected into 100 mL sterile tubes and immediately cooled to 20 °C in a water bath.

Ultrasound treatment was carried out processing 30 mL of sample for 4 min (pulse set to 4 s) through a VC Vibra Cell Ultrasound (US) equipment, model VC 130 working at 130 W/20 kHz (Sonics and Materials Inc., Newtown, CT, USA); the power was set to 60% of the maximum power. Before each treatment, the ultrasonic probe was washed with sterile distilled water and immediately after processing, sample was cooled in ice.

Heat treatment was performed in a water bath previously set to 95 °C for 5 min. Immediately after processing, samples were cooled in ice.

#### 2.3.4. Experiments

*B. clausii* and *B. coagulans* were submitted to chemical, or physical treatments.

- Chemical: Sterile saline solution (0.9% NaCl), supplemented with lemon extract or citrus extract (250 ppm), and inoculated to 5–6 log cfu/mL; each strain was evaluated separately.
- Physical: Sterile saline solution, inoculated to 5–6 log cfu/mL, treated with high homogenization pressure (150 MPa for 1 time; 150 MPa for 2 times, with a rest time of 90 min and 150 MPa for 3 times), ultrasound (power/time/pulse: 60% 4 min 4 s), or heat stress (95 °C for 5 min).

Aliquots of sterile saline solution inoculated with the microbial tests but not treated were used as controls. The viable count was assessed both on the optimal laboratory medium (non-selective medium; Nutrient Agar for *B. coagulans* and Alkaline Nutrient Medium + 0.5% NaCl for *B. clausii*) and on the restrictive one (Nutrient Agar + 0.3% NaCl for *B. coagulans* and Alkaline Agar + 3.0% NaCl for *B. clausii*), after treatment application (T0), 1 day (T1), and 2 days (T2) at room temperature (25 °C). Table 1 shows an overview of the experiments.

#### 2.3.5. Modeling and Statistics

Each experiment was done on two independent batches, i.e., the experiments were performed two times by starting from different spore suspensions/cell suspensions and media prepared in different times. Each time two technical replicates were tested.

The data for the microbiological counts on the optimal media were used to calculate the antimicrobial effect, that is the decrease of the viable count compared to the control sample (not treated microbial suspension).

These values were analyzed through the Multifactorial Analysis of Variance (MANOVA); the antimicrobial effect was the dependent variable, were the independent factors (or categorical predictors) were (in the bracket the qualitative value they could assume): Strain (*B. coagulans* or *B. clausii*), time of sampling (0, or 2), targets (spores, old spores, activated spores, cells), treatment (US, lemon extract, citrus extract, heat). The *post-hoc* test for statistic was Tukey’s test (*p* < 0.05).

After the evaluation of the antimicrobial effect, for each treatment and sample the microbiological counts on optimal and restrictive media were analyzed through *t*-student test (*p* < 0.05); if these values were significantly different, the Percentage of Sub-lethal Injury (*S.I.*, %) was evaluated as follows:

(1) $$S.I.\left(\%\right)=\frac{NSM-SM}{NSM}\;\times \;100.$$

*NSM*: Non selective medium (*NSM*); *SM*; culture medium supplemented salt (3.0% *B. clausii* and 0.3% *B. coagulans*).

Statistic was done through the software Statistica for Windows ver. 10.0 (Statsoft, Tulsa, Okhla.).

### 2.4. Injury Characterization

The kind of injury was assessed on both fresh and old spores treated through ultrasound, heating, citrus and lemon extract; the analyses were done immediately after stress application (T0) and after 2 days (T2) to assess the following parameters: (1) leakage of UV-absorbing substances, (2) BSA protein assay, and (3) colorimetric assay for dipicolinic acid (DPA) in bacterial spores.

#### 2.4.1. Leakage of UV-Absorbing Substances

Spores suspensions were centrifuged at 1200× *g* for 10 min and the UV absorbance of the supernatant was measured at 260 nm and at 280 nm with a spectrophotometer (spectrophotometer UV-VIS DU 640 Beckman (Fullerton, CA, USA) [17].

#### 2.4.2. BSA Protein Assay

Spore suspensions were centrifuged at 1200× *g* for 10 min. Then, 0.1 mL of supernatant was mixed with 2.0 mL of BCA Working Reagent (BCA Protein Assay Reagent Kit, Sigma-Aldrich), and the mixture was incubated at 60 °C for 15 min. The absorbance at 562 nm was then measured with a spectrophotometer. The BCA protein assay was carried out using bovine serum albumin (BSA) as the calibration standard [18].

#### 2.4.3. DPA Protein Assay

After the treatments, 5 mL of spore suspension were thermally treated for 15 min at 121 °C. Then, the suspension was cooled, acidified with 0.1 mL of 1.0 N acetic acid, and left at room temperature for 1 h. Upon centrifugation at 1000× *g* for 10 min, 4 mL of supernatant were put into a clean test tube. One milliliter of freshly prepared reagent, consisting of 1% of Fe(NH_4_)_2_(SO_4_)_2_·6H_2_0 (Sigma-Aldrich) and 1% of ascorbic acid in 0.5 M sodium acetate buffer solution for optode membranes pH 5.5 (Sigma-Aldrich), was added to the supernatant. The absorbance at 440 nm was then measured with a spectrophotometer within 2 h. The colorimetric assay was carried out using DPA (Sigma-Aldrich) as the calibration standard [19].

#### 2.4.4. Statistic Indices

The experiments were done in duplicate on two independent samples; for each sample two technical replicates were used. Mean and standard deviation were calculated.

## 3. Results and Discussion

### 3.1. Antimicrobial Effect

The first step of this research was aimed at assessing the antimicrobial effects of several treatments, including essential oils, and two alternative physical approaches (homogenization and ultrasound); heating was used as the reference method. However, preliminary experiments revealed that homogenization was not able to exert an antimicrobial effect on spores and the effect on cells was slight, thus it was not further used. Since the aim of this paper was also to study sub-lethal injury, the treatments were set to sub-lethal levels to have a fraction of surviving population.

After the treatment, the results were standardized and analyzed through MANOVA to allow a comparison among treatments, strains, and targets. Table 2 shows the standardized effects of the categorical predictors; strain, treatment, time, and target were all significant as individual terms with a different statistical weight. The most important factor was microorganism (*F* = 1689.04), followed by treatment (*F* = 947.03), time (*F* = 66.78), and target (*F* = 55.62), while the most significant interactive terms were “target × treatment” (*F* = 38.26) and “microorganism × target” (*F* = 35.20).

This approach, through the table of the standardized effects, offers a qualitative estimation of each factor (strains, treatment, time, and target), but it could not give an overview on the quantitative effect of each variable, that is how much each predictor affects the dependent variable (antimicrobial effect).

**Figure 1:**
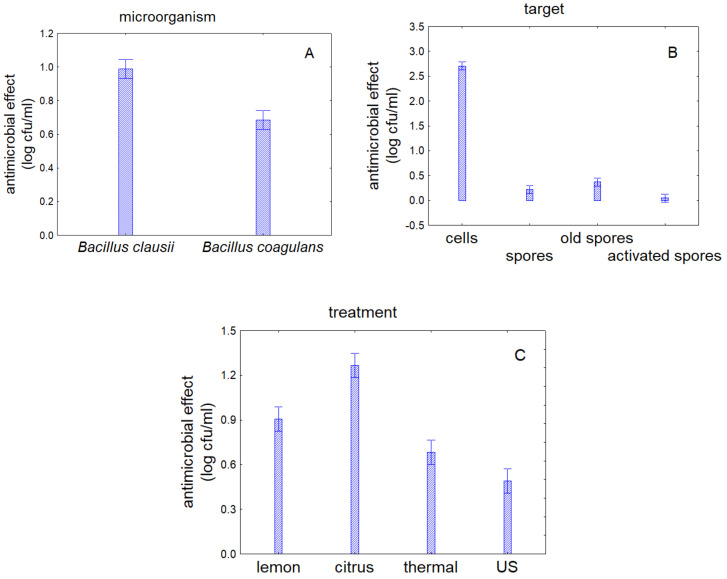
Decomposition of the statistical hypothesis for the effect of individual terms. Bars denote 95% confidence intervals. (**A**) effect of the strain; (**B**) effect of the target; and (**C**) effect of the treatment.

This information can be easily recovered through the decomposition of the statistical hypothesis on both individual and interactive terms. Figure 1 shows the decomposition of the statistical hypothesis for the individual terms of strains (A), target (B), and treatment (C); it is important to point out that a picture of decomposition does not show actual trend, but it is a statistical artifact showing the mathematical and quantitative effect of each variable by excluding all other factors.

Generally, *Bacillus clausii* was less resistant, as statistic evaluated a general antimicrobial effect of 1.0 log cfu/mL vs. 0.75 log cfu/mL of *Bacillus coagulans* (1A); focusing on the target, cells were strongly affected (antimicrobial effect of 2.7 log cfu/mL) and spores appeared resistant (1B). Focusing on the treatment, citrus extract exerted the highest antimicrobial effect (1.2 log cfu/mL) and US the lowest one (0.5 log cfu/mL) (1C).

**Figure 2:**
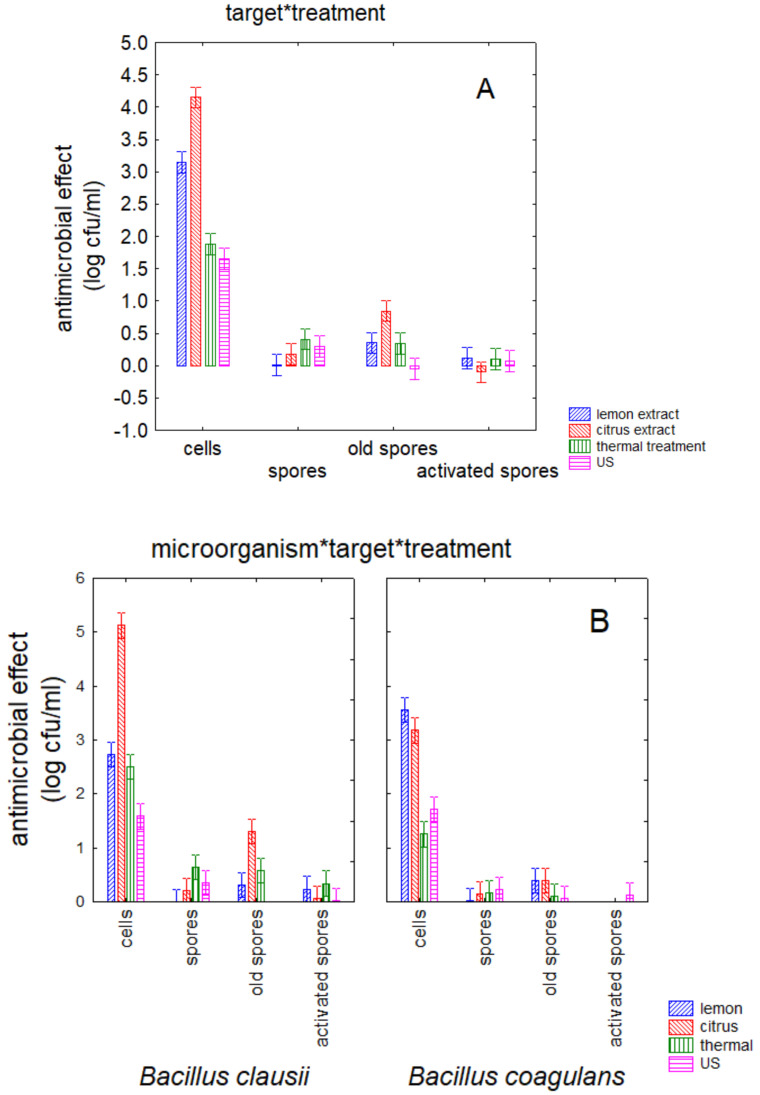
Decomposition of the statistical hypothesis for the effect of interactive terms. Bars denote 95% confidence intervals. (**A**) target × treatment; (**B**) microorganisms × target × treatment.

Figure 2 shows the decomposition of the statistical hypothesis for the interactive terms; the existence of interactive terms suggests that the trends of each categorical predictor could be enhanced or decreased by other factors. In fact, the decomposition of interactive terms is important because it could highlight what happens in real conditions. Figure 2A points out the different effect of the treatments on the targets and suggests that the effect of a treatment could change as a function of targets (cells, fresh, old, or activated spores). This figure confirms the higher susceptibility of cells, but it also highlights that also on cells the treatment had a different effect. In fact, the strongest effect was found for citrus extract (mean antimicrobial effect, 4.2 log cfu/mL), followed by lemon extract (3.2 log cfu/mL) and by the physical treatments (1.7–1.9 log cfu/mL). Figure 2B shows the interaction treatment × strain × target. This kind of decomposition is important because it confirms the different susceptibility of the two strains, depending on the treatment and the targets. A higher bioactivity of citrus extract on the cells of *B. clausii* (5.1 log cfu/mL) compared to *B. coagulans* (3.3 log cfu/mL) was found, as well as the significant effect of citrus extract on the old spores of *B. clausii* (1.2 log cfu/mL).

Many authors reported in the past the suitability of lemon extract as a natural preservative for the inhibition of a wide range of microorganisms [20,21]. Similarly, citrus extract showed promising results in term of juice stabilization [22]. Differently from lemon extract, it is colorless and odorless and water-soluble.

As expected spores were resistant to physical treatments. US could destroy or reduce bacterial clusters and exert an antimicrobial effect through mechanical, physical, and chemical effects of acoustic cavitation [23]. Cavitation is due to growth and subsequent collapse of microscopic bubbles when ultrasonic waves travel through a liquid. Another mode of action was probably related to the formation of free radicals, which might induce adverse chemical changes such as DNA or protein denaturation [24].

A unifying mechanism, called sonoporation, was proposed by Ojha et al. [25]; it relies on the effect of US on cell membrane with six different modes of action. However, this mechanism could probably explain why US were less effective on spores: Sonoporation requires an active membrane as a target, while spores have a different outer layer, thus they were resistant.

### 3.2. Sub-Lethal Injury and Injury Characterization

Hayley and Palombo [26] studied the effects of some EOs towards the spores of *Bacillus subtilis*; they used SEM analysis (Scanning Electron Microscopy) and found that spores treated with EOs appeared withered and deflated with ridges and this effect was more pronounced after a longer exposure time. Thus, they postulated that EOs caused a loss of intracellular material. Cox et al. [27] suggested that tea tree oil components were able to disrupt their structure by increasing fluidity. Cortezzo et al. [28] studied the mode of action of some compounds towards spores and found that few chemicals (like o-chlorocresol) acted on nutrient receptors involved in germination.

On the other hand, ultrasound could disaggregate bacterial clusters and inactivate bacteria through mechanical, physical, and chemical effects [23].

However, there are few evidences on the effects of these treatments on the surviving cells/microorganisms. Table 3 shows the extent of sub-lethal injury; the cells of *B. coagulans* were mainly affected by citrus (injury of 44.50%) and lemon extracts (injury of 33.37%). Also, thermal treatment and ultrasound caused a sub-lethal injury on spores and old spores. On the other hand, EOs did not affect or slightly affect *B. clausii* whilst thermal and ultrasound injured cells and old spores.

**Figure 3:**
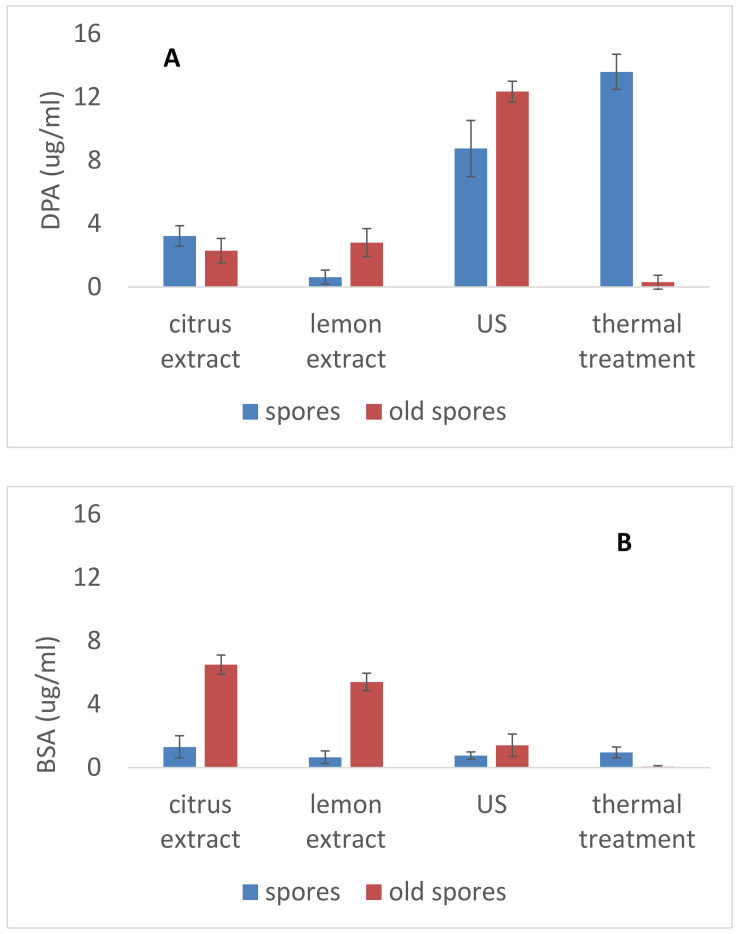
Dipicolinic acid (DPA) (**A**) and protein release (bovine serum albumin (BSA), (**B**) from *B. coagulans* spores immediately after the treatment. Mean ± standard deviation.

After the screening step, some combinations were chosen to study which kind of damage occurred on spores. In this study spores of bacilli released DPA mainly after ultrasound and thermal treatments (Figure 3A), whereas the main sign of injury after the application of lemon and citrus extract was the release of proteins (Figure 3B).

Injured cells often lose some cellular components, like amino acids, 260 nm absorbing material (nucleic acids), and 280 nm absorbing material (protein) through leakage into their surroundings [29,30], since the cell membrane appears to be the component most commonly affected [31]. However, it is a matter of debate if the release of DPA is a sign of injury or not; DPA release, in fact, is associated to spores’ germination after the activation of the nutrient receptors. However, measurement of DPA could be used as a potential signal indicator of spore injury [32], as also reported by Chaves Lopèz et al. [19], who defined DPA release as a sign of spore irreversible damage under the most severe pressurization conditions.

The release of DPA from the spore core during a thermal treatment is still under discussion and has not been evaluated in full detail [33]. Primarily, the high amount of DPA coupled with the low amount of water is responsible for the wet heat resistance. The most likely mechanisms for spore inactivation by heat are a rupture of the spore’s inner membrane and the inactivation of core enzymes [34,35].

## 4. Conclusions

The results of this paper show the effects of some chemical and physical treatments on the spores of two strains of *Bacillus* as model microorganisms to study the sub-lethal injury on cells and spores; in particular, lemon and citrus extracts exerted a strong antibacterial action on cells. Moreover, the age of spores was less significant for the antimicrobial effect.

Both essential oils and physical treatments could cause a sub-lethal injury on the surviving cells and spores, but it was not possible to point out a general trend because the results were strongly strain dependent; after the treatments, the spores of *B. coagulans* experienced the release of proteins and DPA, but further investigations are required to elucidate the general mechanism beyond this effect.

In conclusion, this paper shows that the spores of *Bacillus* spp. could experience an injury when treated by chemical or physical treatments; this phenomenon should be considered when the optimization of a treatment is carried out, because injured microorganisms could either repair the damage or be inactivated with a different effect on the microbial stability of foods.

## Figures and Tables

**Table 1:** Overview of the experiments.

Treatments	Conditions
Lemon extract	Saline solution supplemented with lemon extract (250 ppm)
Citrus extract	Saline solution supplemented with citrus extract (250 ppm)
Heating	Saline solution treated at 95 °C for 5 min
Ultrasound	Saline solution treated through US at a power of 60% for 4 min; pulse set to 4 s
Homogenization	Saline solution treated at 150 MPa for 1 time
	Saline solution treated at 150 MPa for 2 times
	Saline solution treated at 150 MPa for 3 times
Targets and experiments	Each treatment was tested on spores, old spores, activated spores, and cells; thus, 20 experiments were done (5 treatments × 4 targets)
Analysis	The microbiological analyses were done immediately after the treatment (T0), and after 1 (T1) or 2 days (T2); counts were done on two media: Optimal media and restrictive media (the details are in the text)

**Table 2:** Standardized effect of microorganism (*Bacillus clausii* or *B. coagulans*), treatment (citrus or lemon extract, US or thermal treatment), time (immediately after the treatment and after 2 days) and target (cells, spores, old spores, and activated spores) on the antimicrobial effect (decrease of viable count, log cfu/mL). SS, sum of square residual; MS, mean square residual.

	SS	Degree of Freedom	MS	Fisher Test
Microorganism (1)	89.76	1	89.76	1689.04
Target (2)	2.96	1	2.96	55.62
Treatment (3)	150.99	3	50.33	947.03
Time (4)	10.65	3	3.55	66.78
Microorganism × target	1.87	1	1.87	35.20
Microorganism × treatment	0.75	3	0.25	4.71
Target × treatment	6.10	3	2.03	38.26
Microorganism × time	- ^1^	-	-	-
Target × time	0.11	1	0.11	2.10
Treatment × time	0.77	3	0.26	4.86
Microorganism × target × treatment	0.46	3	0.15	2.90
Microorganism × target × time	5.78	9	0.64	12.08
Microorganism × treatment × time	0.48	3	0.16	2.99
Target × treatment × time	0.86	3	0.29	5.37
1 × 2 × 3 × 4	1.16	9	0.13	2.42
Error	1.87	9	0.21	3.92

^1^ Not significant.

**Table 3:** Sub-lethal injury (%) on *B. coagulans* and *B. clausii* after the addition of citrus or lemon extract or the application of US or a thermal treatment. Mean values ± standard deviation.

*B. coagulans*					
**Treatment**	**Time (day)**	**Cells**	**Spores**	**Old Spores**	**Activated Spores**
Lemon extract	0	- ^1^	-	4.85 ± 0.40	-
	2	33.37 ± 3.82	-	-	-
Citrus extract	0	44.50 ± 10.27	-	-	-
	2	-	-	-	18.27 ± 0.33
Thermal treatment	0	-	4.45 ± 0.11	2.40 ± 0.40	-
	2	-	10.51 ± 0.90	-	-
US	0	19.20 ± 3.11	-	3.61 ± 0.13	-
	2	-	-	-	-
*B. clausii*					
**Treatment**	**Time (day)**	**Cells**	**Spores**	**Old Spores**	**Activated Spores**
Lemon extract	0	-	-	-	-
	2	-	-	-	-
Citrus extract	0	/ ^2^	-	-	-
	2	/	-	7.77 ± 1.67	-
Thermal treatment	0	-	-	7.72 ± 0.41	-
	2	24.08 ± 6.45	-	-	-
US	0	24.50 ± 1.15	-	15.69 ± 0.94	-
	2	39.54 ± 2.81	-	-	-

^1^ No injury detected. ^2^ Not assessed, because the viable count on the optimal medium was below the detection limit.
